# Supplementary material for: Peripheral modulation of Pumilio in intestinal stem cells and the corpus allatum affects sleep latency in Drosophila
Source: PLoS One. 2026 Apr 8;21(4):e0337303. doi: 10.1371/journal.pone.0337303 (PMC13061178; doi:10.1371/journal.pone.0337303)
Supplement: S1 File — (DOCX) [file pone.0337303.s001.docx]

**Supporting Information**

| **Fig. #** | **Genotype** |
| --- | --- |
| Fig. 2 | w^1118^/y^1^,v^1^; esg-Gal4, UAS-2xYFP/+; Su(H)GBE-Gal80, tub-Gal80ts/UAS-GFP  where UAS-GFP = P{y[+t7.7] v[+t1.8]=UAS-GFP.VALIUM10}attP2  w^1118^/y^1^,v^1^; esg-Gal4, UAS-2xYFP/+; Su(H)GBE-Gal80, tub-Gal80ts/UAS-pum^RNAi^  where UAS-pum^RNAi^ = P{y[+t7.7] v[+t1.8]=TRiP.JF02267}attP2 |
| Fig. 3a-b | w^1118^; esg-Gal4, UAS-2xYFP/+; Su(H)GBE-Gal80, tub-Gal80ts/UAS-RpL3.FLAG  w^1118^; esg-Gal4, UAS-2xYFP/+; Su(H)GBE-Gal80, tub-Gal80ts/UAS-pum, UAS-RpL3.FLAG |
| Fig. 3c-d | w^1118^; CyO/+; Su(H)GBE-Gal80, tub-Gal80ts/UAS-pum  w^1118^; esg-Gal4, UAS-2xYFP/+; Su(H)GBE-Gal80, tub-Gal80ts/UAS-pum |
| Fig. 4  and Fig. S1 | w^1118^/y^1^,sc*,v^1^;sev^21^; esg-Gal4, UAS-2xYFP/+; Su(H)GBE-Gal80, tub-Gal80ts/UAS-mCherry^RNAi^  where UAS-mCherry^RNAi^ =P{y[+t7.7] v[+t1.8]=VALIUM20-mCherry.RNAi}attP2  w^1118^/y^1^,sc^*^,v^1^,sev^21^; esg-Gal4, UAS-2xYFP/+; Su(H)GBE-Gal80, tub-Gal80ts/UAS-pum^RNAi^  where UAS-pum^RNAi^ = P{y[+t7.7] v[+t1.8]=TRiP.HMS01564}attP2 |
| Fig. 5a | M{3xGBE-GAL80}ZH-2A, w*/y^1^,sc*,v^1^;sev^21^; P{mira(KDRT.stop)GAL4}attP40, P{tubP-GAL80[ts]}20 ; P{CG10116-KDR.PEST}attP2/UAS-mCherry^RNAi^  where UAS-mCherry^RNAi^ =P{y[+t7.7] v[+t1.8]=VALIUM20-mCherry.RNAi}attP2  M{3xGBE-GAL80}ZH-2A, w*/y^1^,sc*,v^1^;sev^21^; P{mira(KDRT.stop)GAL4}attP40, P{tubP-GAL80[ts]}20 ; P{CG10116-KDR.PEST}attP2/UAS-pum^RNAi^  where UAS-pum^RNAi^ = P{y[+t7.7] v[+t1.8]=TRiP.HMS01564}attP2 |
| Fig. 5b | w*/y^1^,sc*,v^1^,sev^21^; Aug21-Gal4/+; UAS-mCherry^RNAi^/+  where UAS-mCherry^RNAi^ =P{y[+t7.7] v[+t1.8]=VALIUM20-mCherry.RNAi}attP2  w*/y^1^,sc*,v^1^,sev^21^; Aug21-Gal4/+; UAS-mCherry^RNAi^/+  where UAS-pum^RNAi^ = P{y[+t7.7] v[+t1.8]=TRiP.HMS01564}attP2 |
| Fig. 5c | w*; Aug21-Gal4/+; P{w[+mC]=UAS-tdTom.S}3/P{w[+mC]=tubP-GAL80[ts]}2 |
| Fig. S2 | w^1118^/y^1^,v^1^; esg-Gal4, UAS-2xYFP/+; Su(H)GBE-Gal80, tub-Gal80ts/UAS-GFP  where UAS-GFP = P{y[+t7.7] v[+t1.8]=UAS-GFP.VALIUM10}attP2  w^1118^/y^1^,v^1^; esg-Gal4, UAS-2xYFP/+; Su(H)GBE-Gal80, tub-Gal80ts/UAS-pum^RNAi^  where UAS-pum^RNAi^ = P{y[+t7.7] v[+t1.8]=TRiP.JF02267}attP2 |
| Fig. S3 | w^1118^/y^1^,v^1^; esg-Gal4, UAS-2xYFP/+; Su(H)GBE-Gal80, tub-Gal80ts/UAS-GFP  where UAS-GFP = P{y[+t7.7] v[+t1.8]=UAS-GFP.VALIUM10}attP2  w^1118^/y^1^,v^1^; esg-Gal4, UAS-2xYFP/+; Su(H)GBE-Gal80, tub-Gal80ts/UAS-pum^RNAi^  where UAS-pum^RNAi^ = P{y[+t7.7] v[+t1.8]=TRiP.JF02267}attP2 |
| Fig. S4 | w*/y^1^,sc*,v^1^,sev^21^; Aug21-Gal4/+; UAS-mCherry^RNAi^/+  where UAS-mCherry^RNAi^ =P{y[+t7.7] v[+t1.8]=VALIUM20-mCherry.RNAi}attP2  w*/y^1^,sc*,v^1^,sev^21^; Aug21-Gal4/+; UAS-EGFP^RNAi^/+  where UAS-EGFP^RNAi^ =P{VALIUM22-EGFP.RNAi.1}attP2  w*/y^1^,sc*,v^1^,sev^21^; Aug21-Gal4/+; UAS-pum^RNAi^/+  where UAS-pum^RNAi^ = P{y[+t7.7] v[+t1.8]=TRiP.HMS01564}attP2 |
| Fig. S5 | w^1118^/y^1^,sc*,v^1^;sev^21^;CyO/+; Su(H)GBE-Gal80, tub-Gal80ts/UAS-mCherry^RNAi^  where UAS-mCherry^RNAi^ =P{y[+t7.7] v[+t1.8]=VALIUM20-mCherry.RNAi}attP2  w^1118^/y^1^,sc^*^,v^1^,sev^21^; CyO/+; Su(H)GBE-Gal80, tub-Gal80ts/UAS-pum^RNAi^  where UAS-pum^RNAi^ = P{y[+t7.7] v[+t1.8]=TRiP.HMS01564}attP2 |
| Fig. S6 | w^1118^/y^1^,v^1^; esg-Gal4, UAS-2xYFP/+; Su(H)GBE-Gal80, tub-Gal80ts/UAS-GFP  where UAS-GFP = P{y[+t7.7] v[+t1.8]=UAS-GFP.VALIUM10}attP2  w^1118^/y^1^,v^1^; esg-Gal4, UAS-2xYFP/+; Su(H)GBE-Gal80, tub-Gal80ts/UAS-pum^RNAi^  where UAS-pum^RNAi^ = P{y[+t7.7] v[+t1.8]=TRiP.JF02267}attP2 |

**Table S1. Genotypes of flies used.**


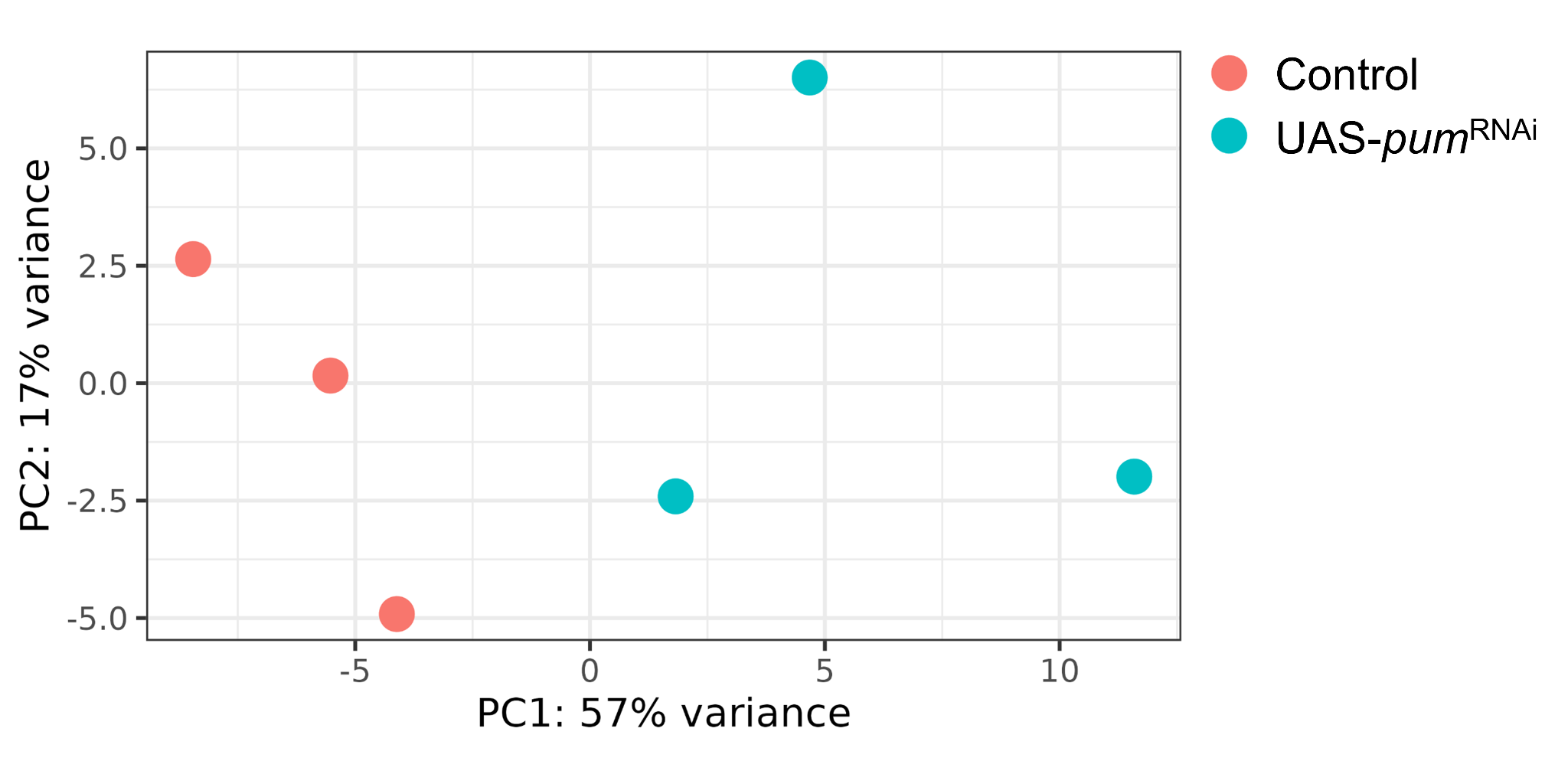


**Figure S1**. Principal Component Analysis (PCA) of transcriptomic data. PCA plot displaying the variation between samples based on genome-wide gene expression profiles. The first principal component (PC1) accounts for 57% of the variance and separates the samples by experimental condition, while the second principal component (PC2) accounts for 17% of the variance. Each point represents an individual biological replicate. Samples are color-coded by condition: Control, UAS-mCherryRNAi (salmon circles; n=3) and UAS-pumRNAi (teal circles; n=3). Following read quality control and trimming, RNA-seq reads were aligned to the Drosophila melanogaster reference genome (dm6) using STAR. Gene expression data was batch-corrected using ComBat-Seq prior to PCA visualization.

**Figure S2. Conditional knockdown of *pumilio* in *esg*-positive cells shows similar trends in sleep latency reduction in female flies.** (a, b) Nighttime total sleep (a) and sleep latency (b) for female flies in independent experimental repeat 1. (c, d) Nighttime total sleep (c) and sleep latency (d) for female flies in independent experimental repeat 2. For both experiments, flies with *pum* knockdown (*esgGal4^ts^, Su(H)Gal80 > UAS-pum^RNAi^*) are compared to control flies (*esgGal4^ts^, Su(H)Gal80 > UAS-GFP*). The shaded region indicates the period when flies were maintained at 29°C to induce transgene expression. Sample sizes were as follows: Repeat 1 (Control, n=32; *pum*^RNAi^, n=31) and Repeat 2 (Control, n=32; *pum^RNAi^*, n=32). Values and error bars are mean ± s.e.m. Asterisks indicate statistically significant differences (* *P* < 0.05, ** *P* < 0.01, *** *P* < 0.001, **** *P* < 0.0001, Mann-Whitney test).
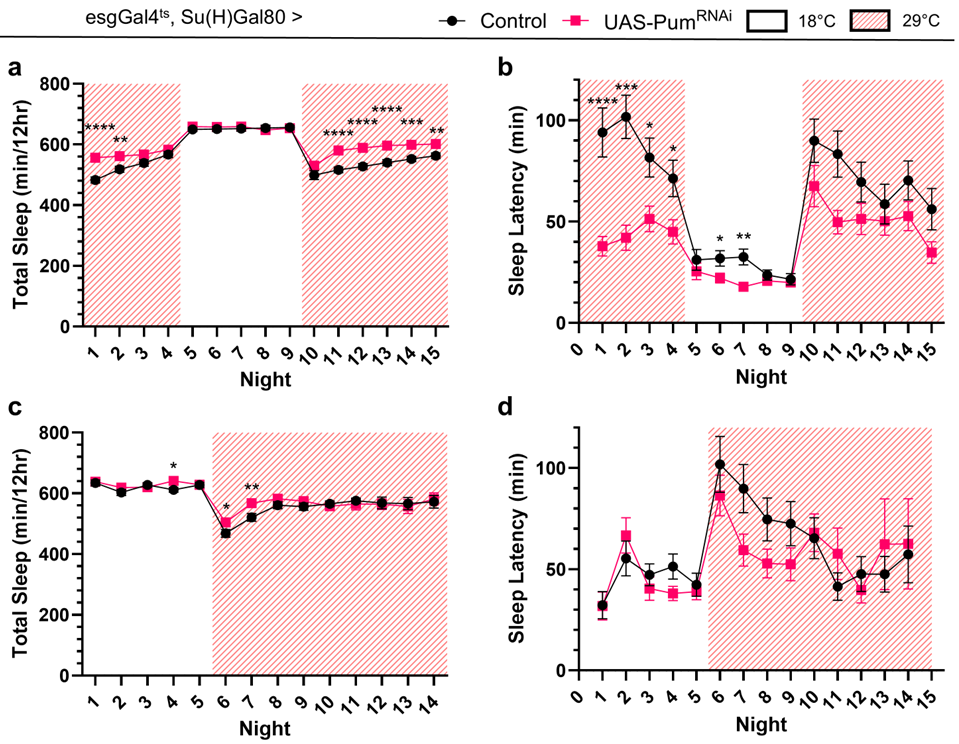


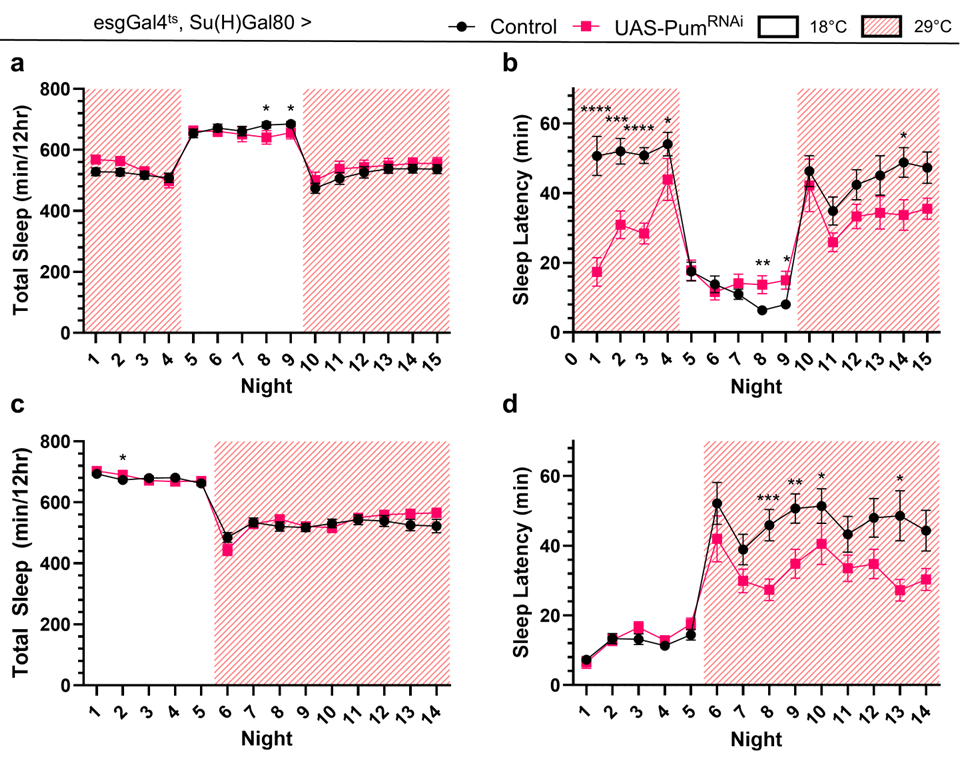


**Figure S3. Independent experimental repeats confirm that conditional knockdown of *pumilio* in *esg*-positive cells reduces nighttime sleep latency in males.** (a, b) Nighttime total sleep (a) and sleep latency (b) for male flies in independent experimental repeat 1. (c, d) Nighttime total sleep (c) and sleep latency (d) for male flies in independent experimental repeat 2. For both experiments, flies with *pum* knockdown (*esgGal4^ts^, Su(H)Gal80 > UAS-pum^RNAi^*) are compared to control flies (*esgGal4^ts^, Su(H)Gal80 > UAS-GFP*). The shaded region indicates the period when flies were maintained at 29°C to induce transgene expression. Sample sizes were as follows: Repeat 1 (Control, n=30; *pum*^RNAi^, n=15) and Repeat 2 (Control, n=32; *pum*^RNAi^, n=32). Values and error bars are mean ± s.e.m. Asterisks indicate statistically significant differences (* *P* < 0.05, ** *P* < 0.01, *** *P* < 0.001, **** *P* < 0.0001, Mann-Whitney test).


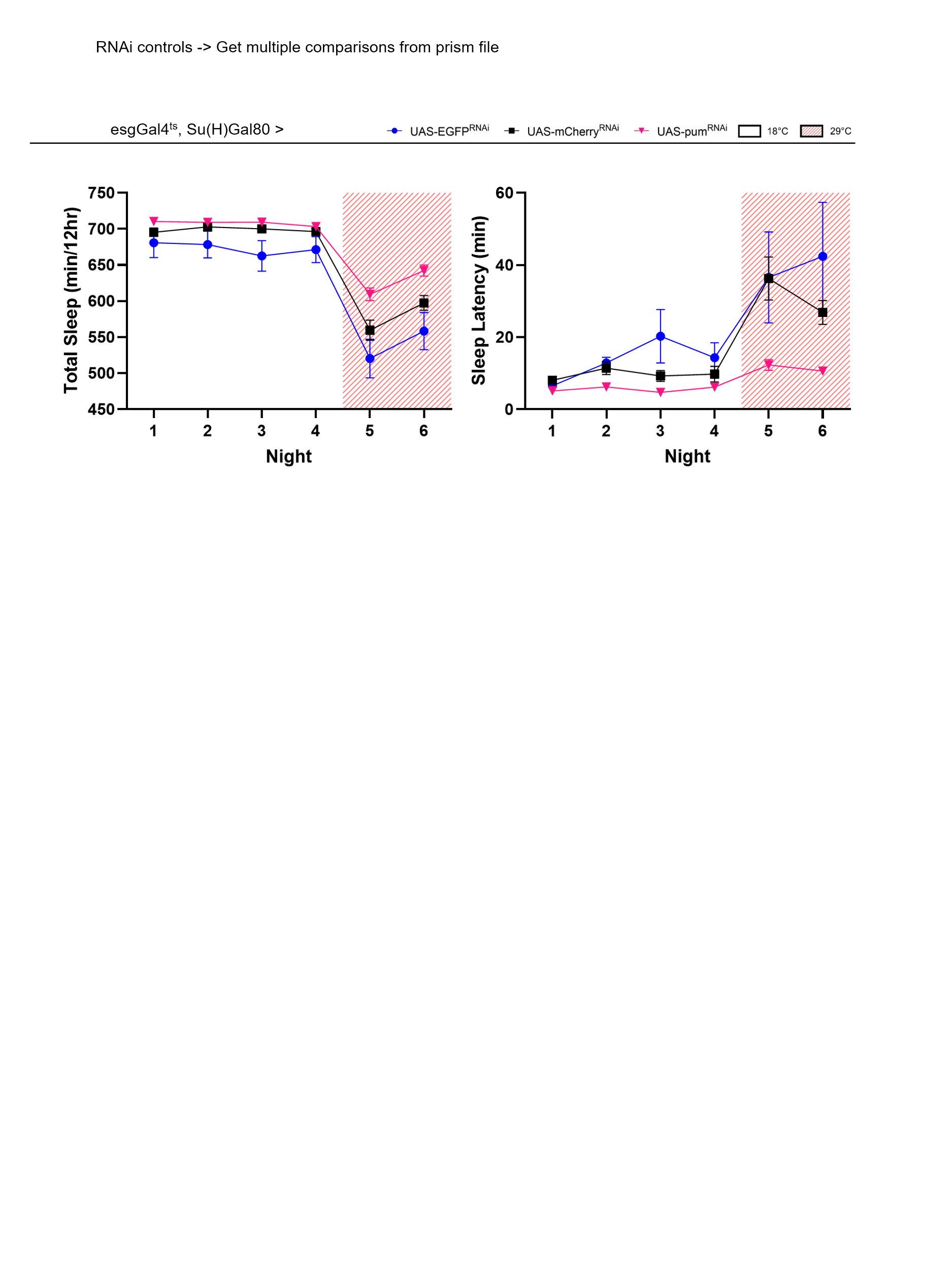


**Figure S4. The sleep latency phenotype is specific to *pumilio* knockdown and not a general effect of RNAi expression.** Nighttime total sleep (left) and sleep latency (right) for male flies comparing *pum* knockdown with two different RNAi controls. The groups shown are: *esgGal4^ts^, Su(H)Gal80 > UAS-EGFP^RNAi^* (VALIUM22, n=32), *esgGal4^ts^, Su(H)Gal80 > UAS-mCherry^RNAi^* (VALIUM20, n=31), and *esgGal4^ts^, Su(H)Gal80 > UAS-pum^RNAi^* (VALIUM20, n=32). The shaded region indicates the period when flies were maintained at 29°C to induce transgene expression. Values are plotted as mean ± s.e.m. Statistical significance was assessed using a mixed-effects model with Geisser–Greenhouse correction, followed by Tukey’s multiple comparisons test; full statistical results are provided in Supplementary Table S2.


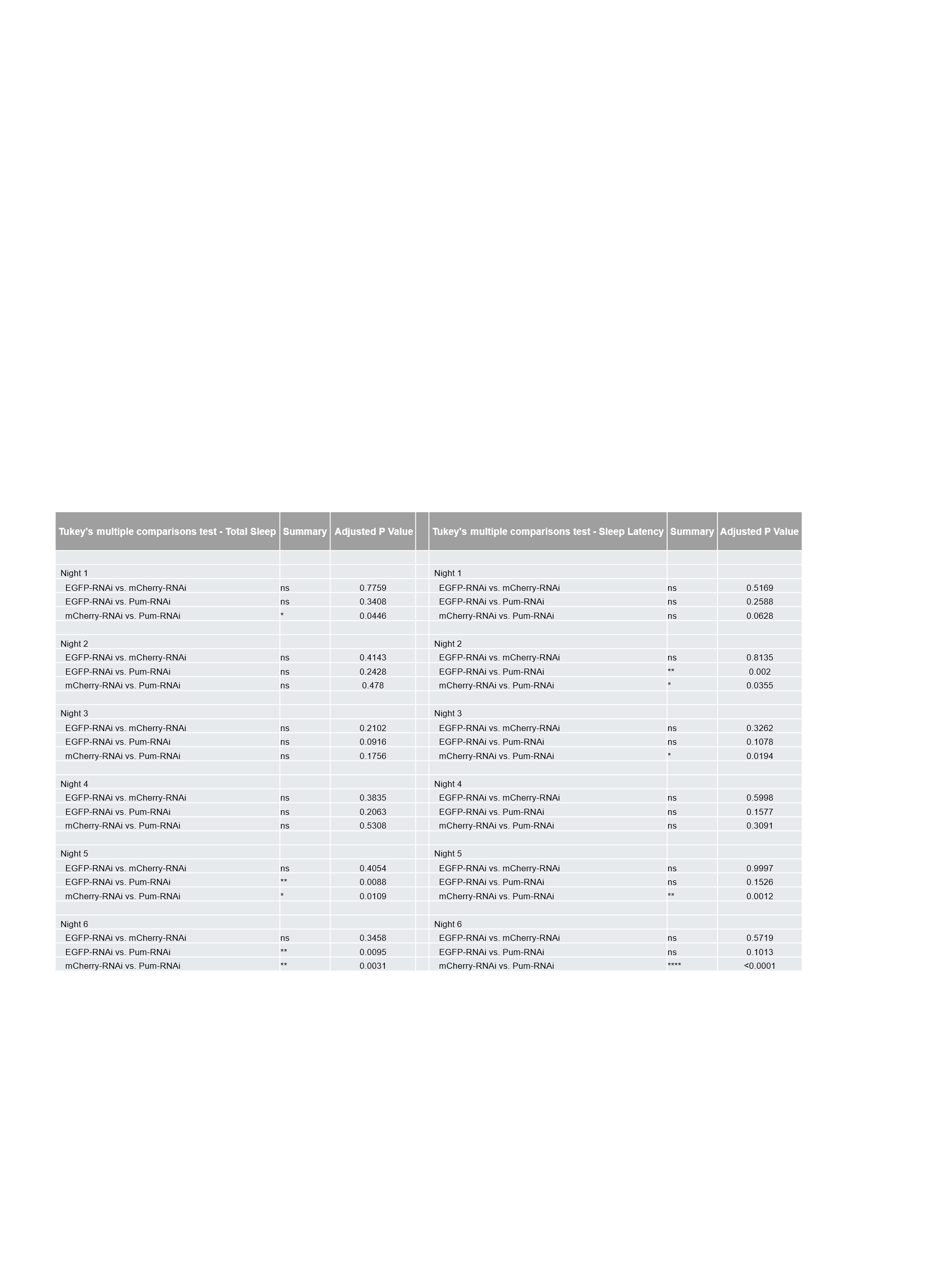


**Table S2. Statistical analysis for RNAi control experiments shown in Supplementary Figure S4.** Tukey's multiple comparisons test results for nighttime total sleep and sleep latency for each night of the experiment shown in Supplementary Fig. S4. Adjusted *P* values are displayed for each pairwise comparison (ns *P* > 0.05, * *P* < 0.05, ** *P* < 0.01, *** *P* < 0.001, **** *P* < 0.0001).


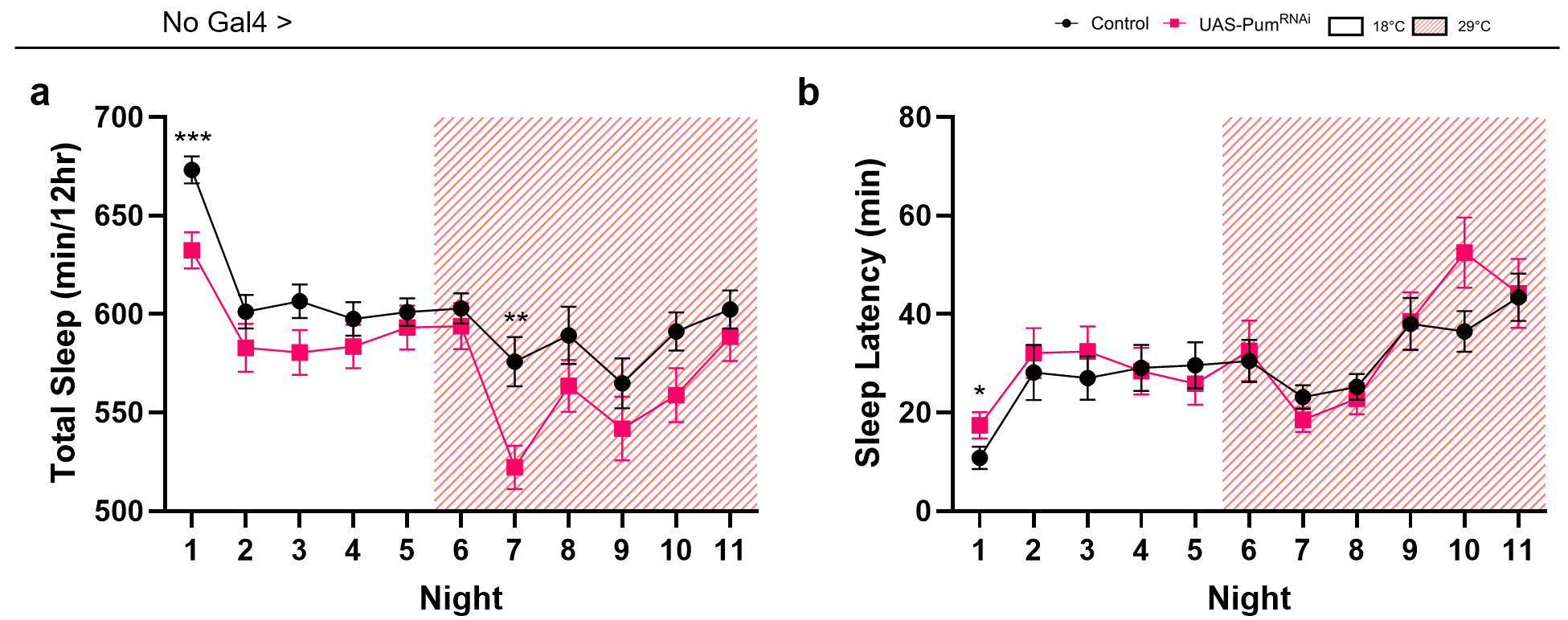


**Figure S5. The sleep latency phenotype is dependent on Gal4-mediated transgene expression.** (a) Nighttime total sleep and (b) nighttime sleep latency for male flies lacking a Gal4 driver. Control flies *UAS-mCherry^RNAi^* (*CyO / + ; UAS-mCherry^RNAi^ / su(H)GBE-Gal80, tubGal80ts*) were compared *UAS-pumRNAi* flies (*CyO / + ; UAS-pum^RNAi^/su(H)GBE-Gal80, tubGal80ts*). The lack of a consistent, significant difference in sleep latency between the groups at 29°C (shaded region) demonstrates that the phenotype is not caused by leaky transgene expression in the absence of the driver. Values are plotted as mean ± s.e.m. Asterisks indicate statistically significant differences between genotypes on a given night (*P* < 0.05, * *P* < 0.01, ** *P* < 0.001, *** *P* < 0.0001, Mann-Whitney test).


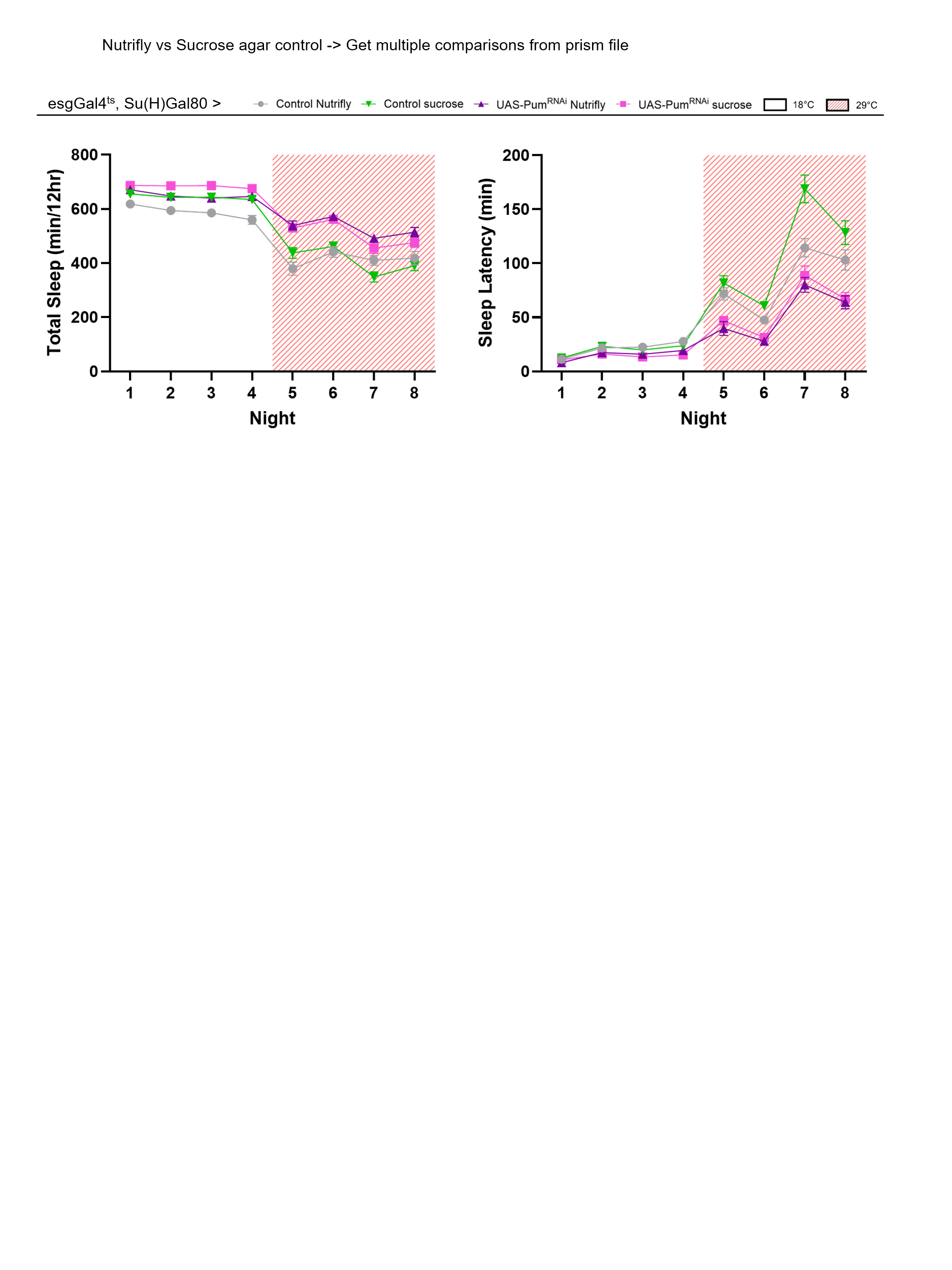


**Figure S6. The effect of peripheral *pumilio* knockdown on sleep latency persists regardless of dietary conditions.** (a) Nighttime total sleep and (b) nighttime sleep latency for male flies maintained on either a standard Nutrifly diet or a sucrose-only diet. Four groups are compared: Control flies (*esgGal4^ts^, Su(H)Gal80 > UAS-GFP*) on Nutrifly (n=28) or sucrose (n=31), and *pum* knockdown flies (*esgGal4^ts^, Su(H)Gal80 > UAS-pum^RNAi^*) on Nutrifly (n=27) or sucrose (n=30). The shaded region indicates the period when flies were maintained at 29°C to induce transgene expression. Values are plotted as mean ± s.e.m. Statistical significance was assessed using a mixed-effects model with Geisser–Greenhouse correction, followed by Tukey’s multiple comparisons test; full statistical results are provided in Supplementary Table S3.


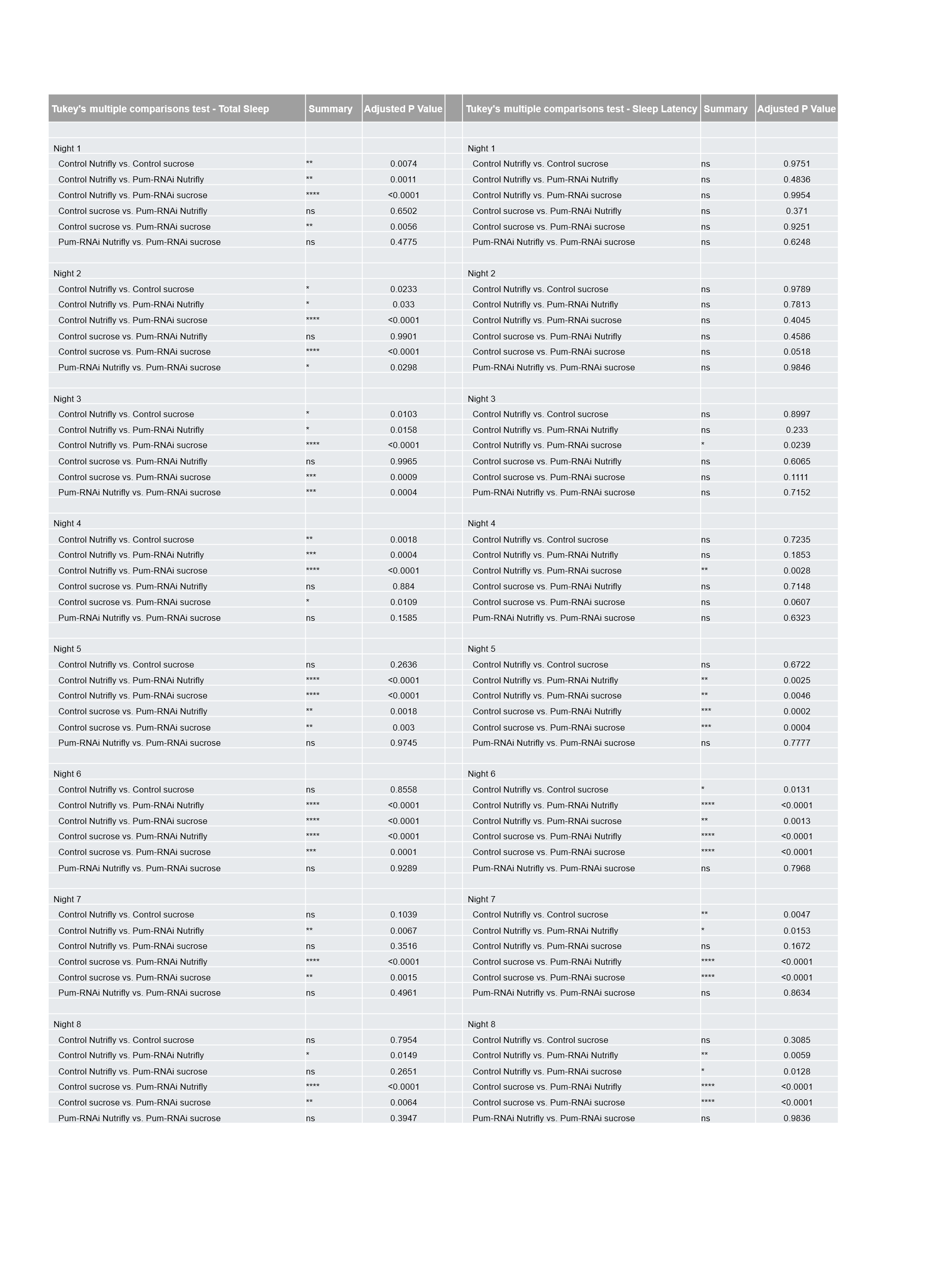


**Table S3. Statistical analysis of dietary effects on sleep parameters shown in Supplementary Figure S6.** Tukey's multiple comparisons test results for nighttime total sleep and sleep latency for each night of the experiment shown in Supplementary Fig. S6. Adjusted *P* values are displayed for each pairwise comparison (ns *P* > 0.05, * *P* < 0.05, ** *P* < 0.01, *** *P* < 0.001, **** *P* < 0.0001).


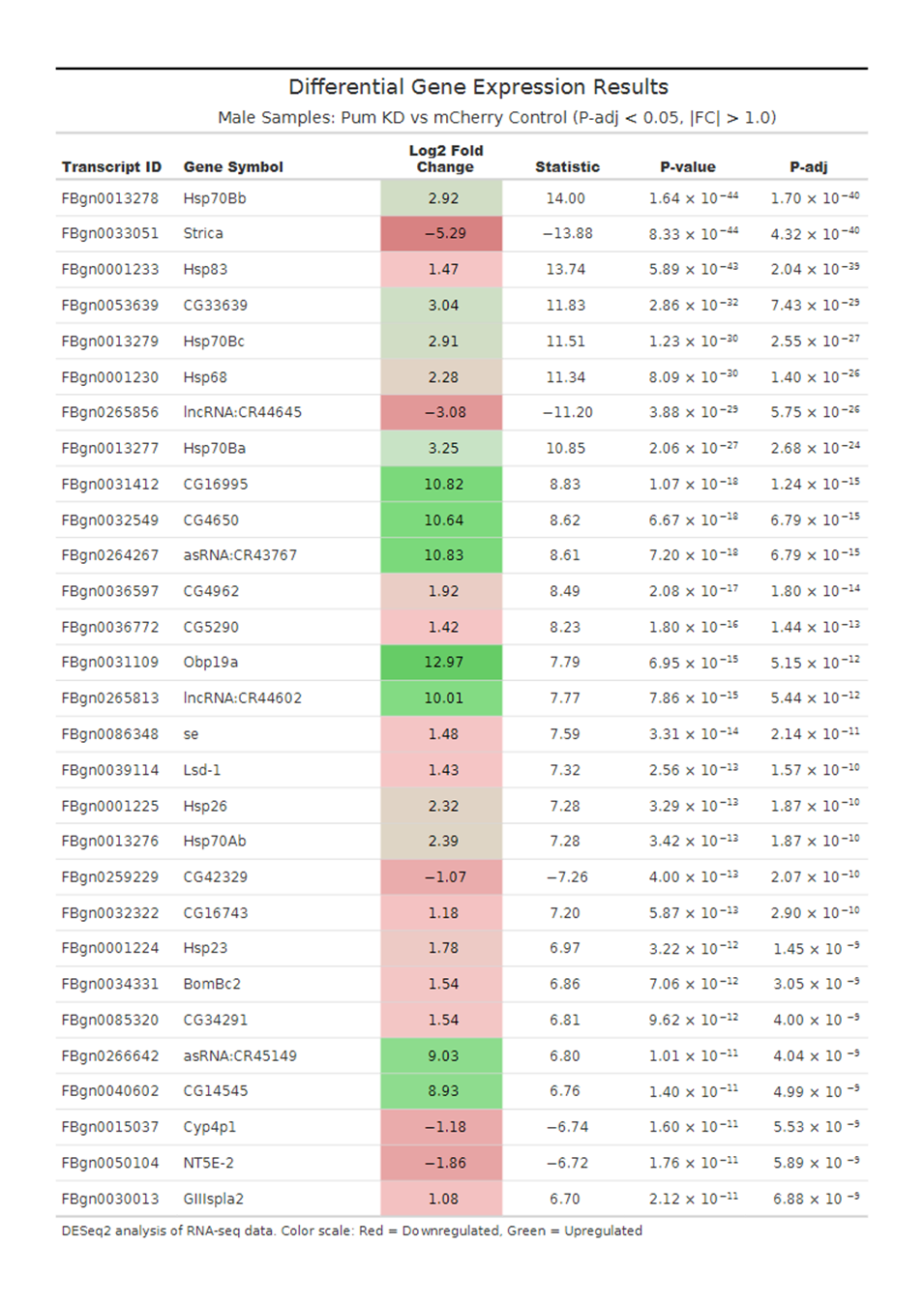

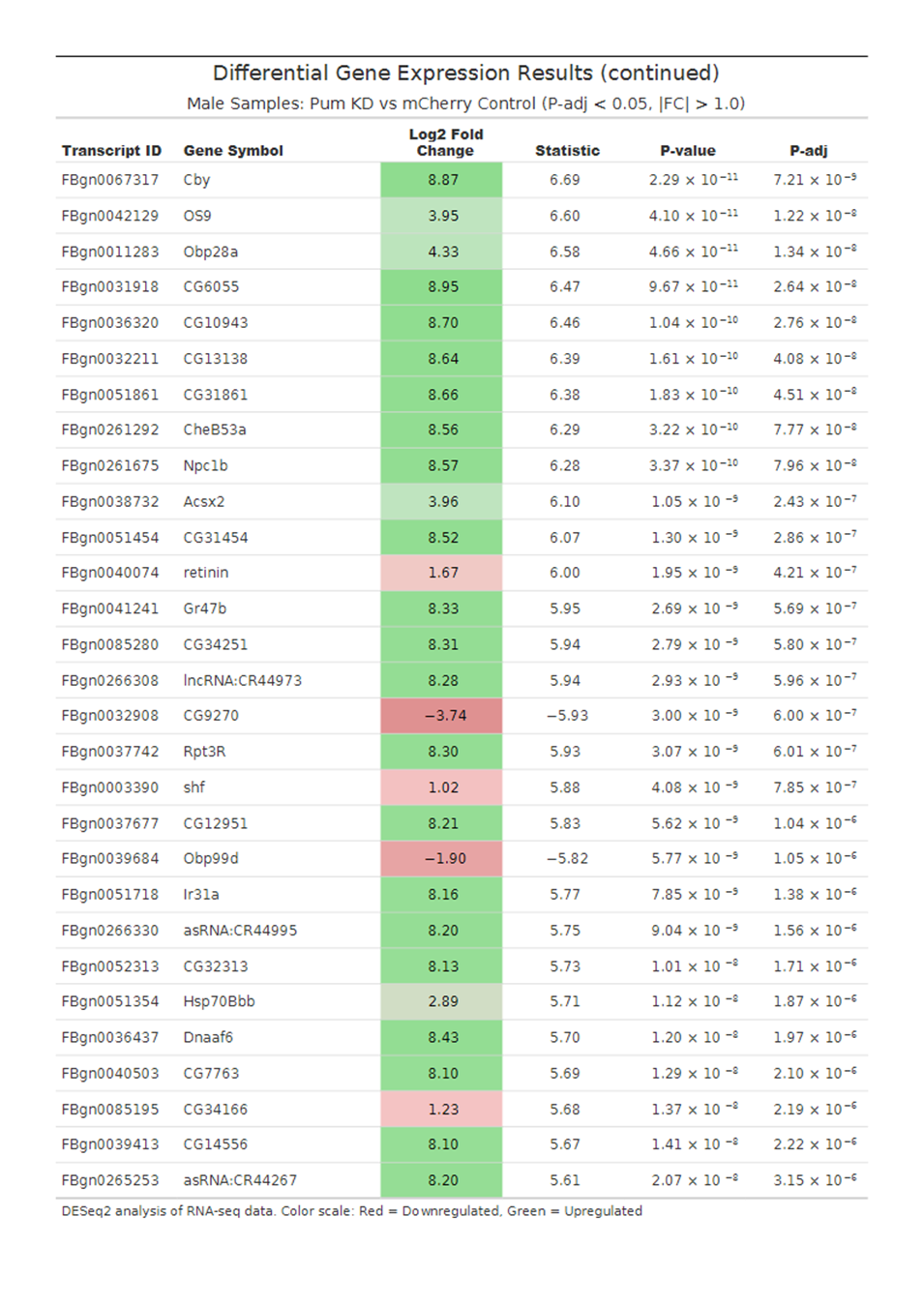

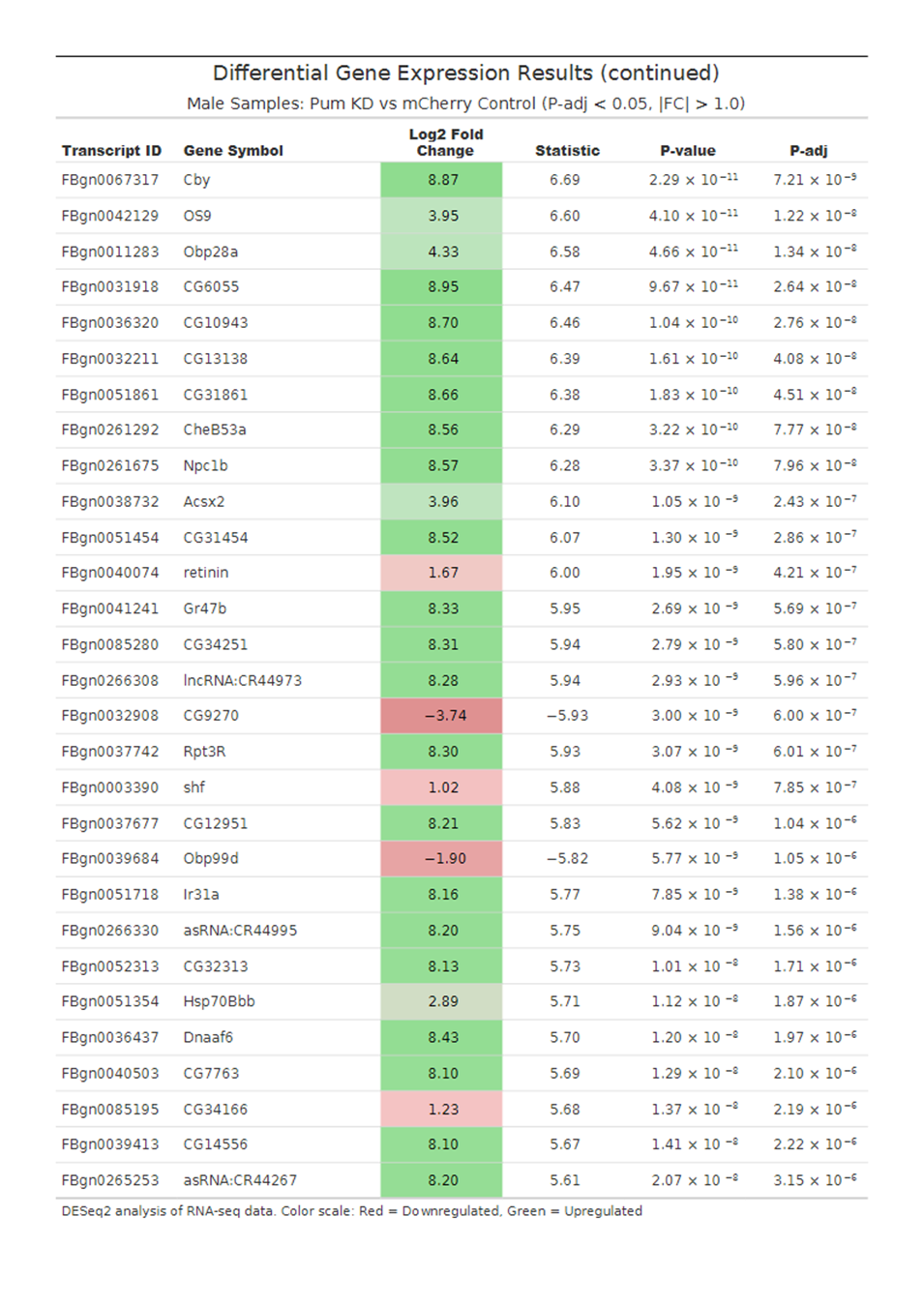

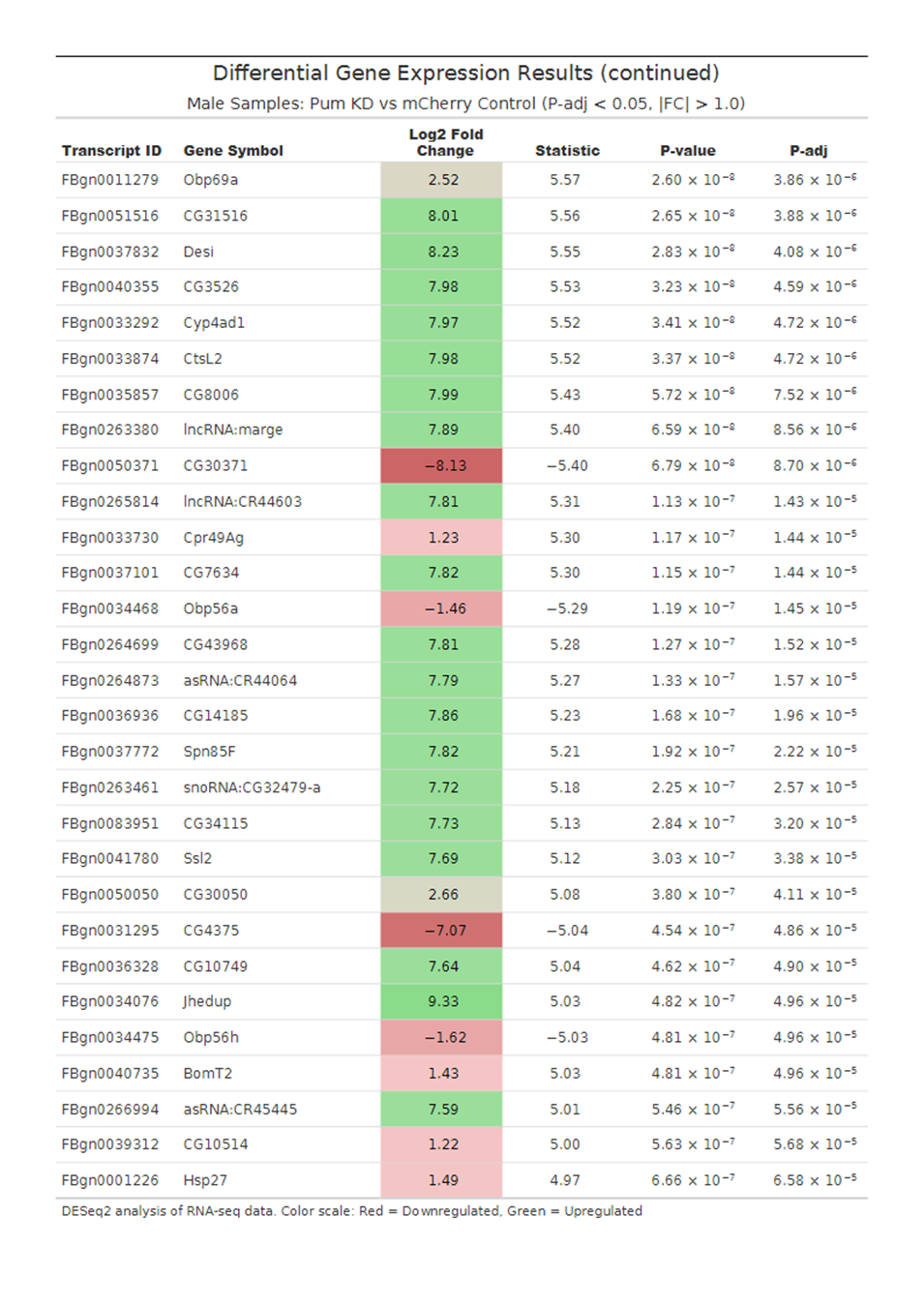

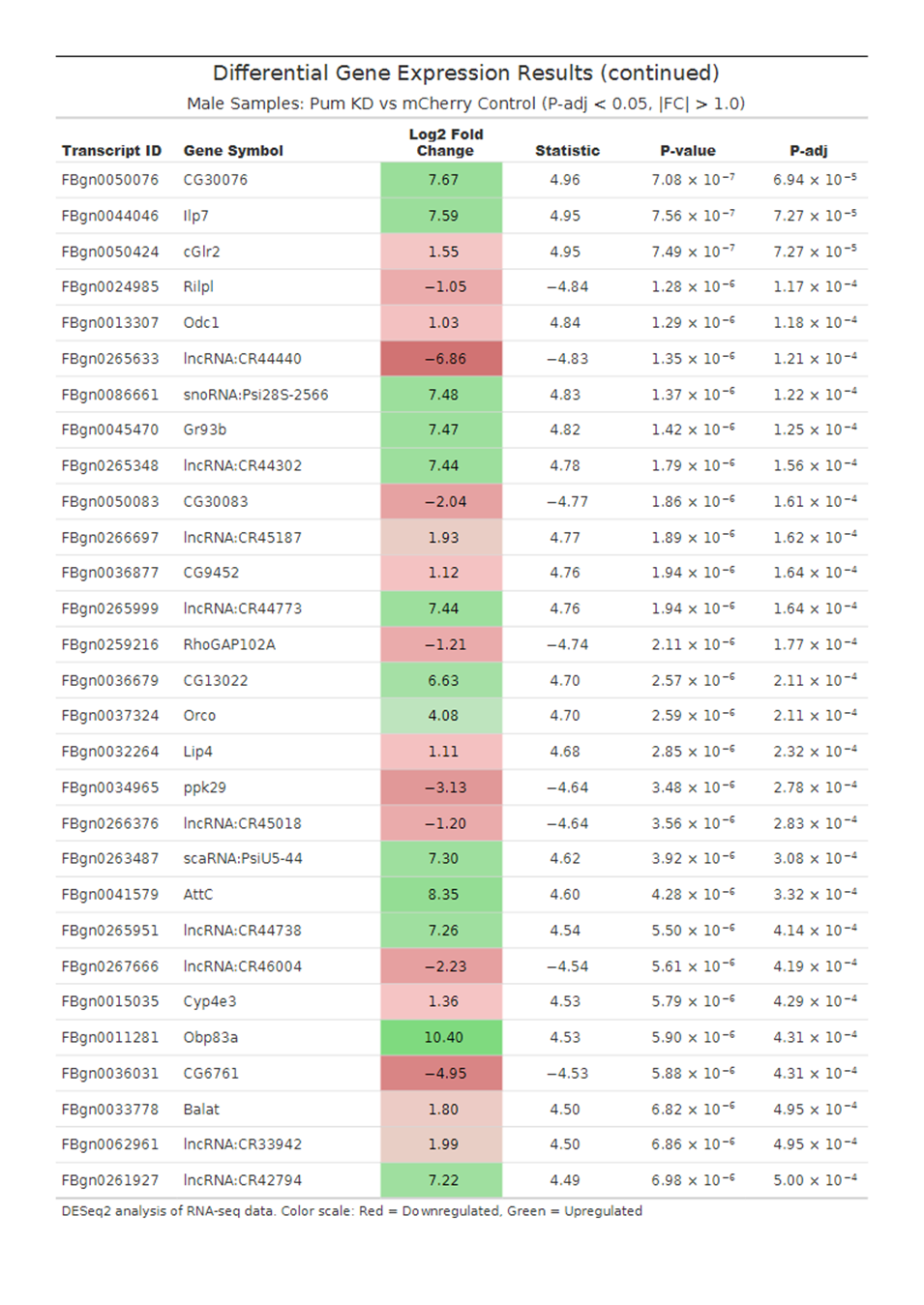

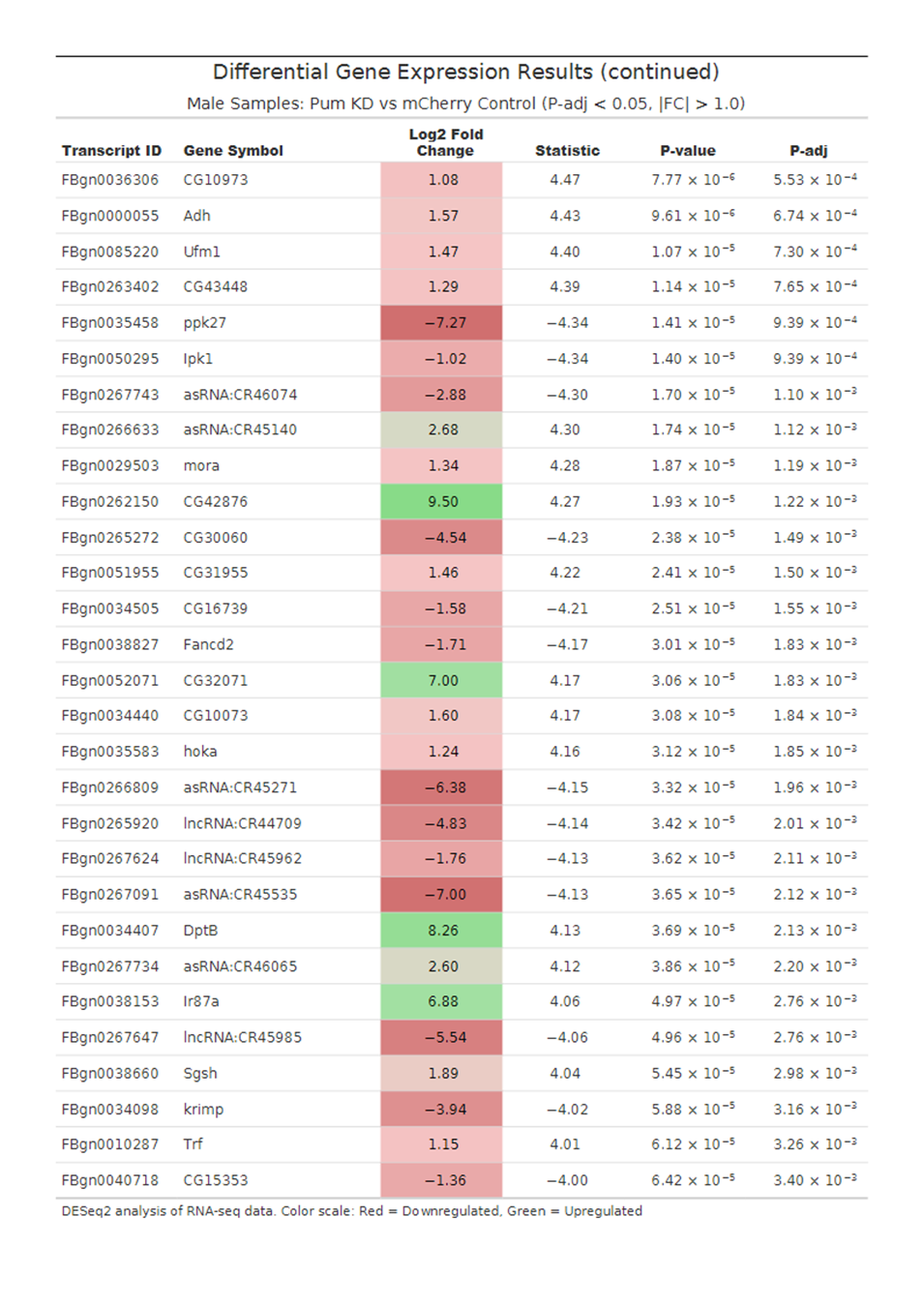

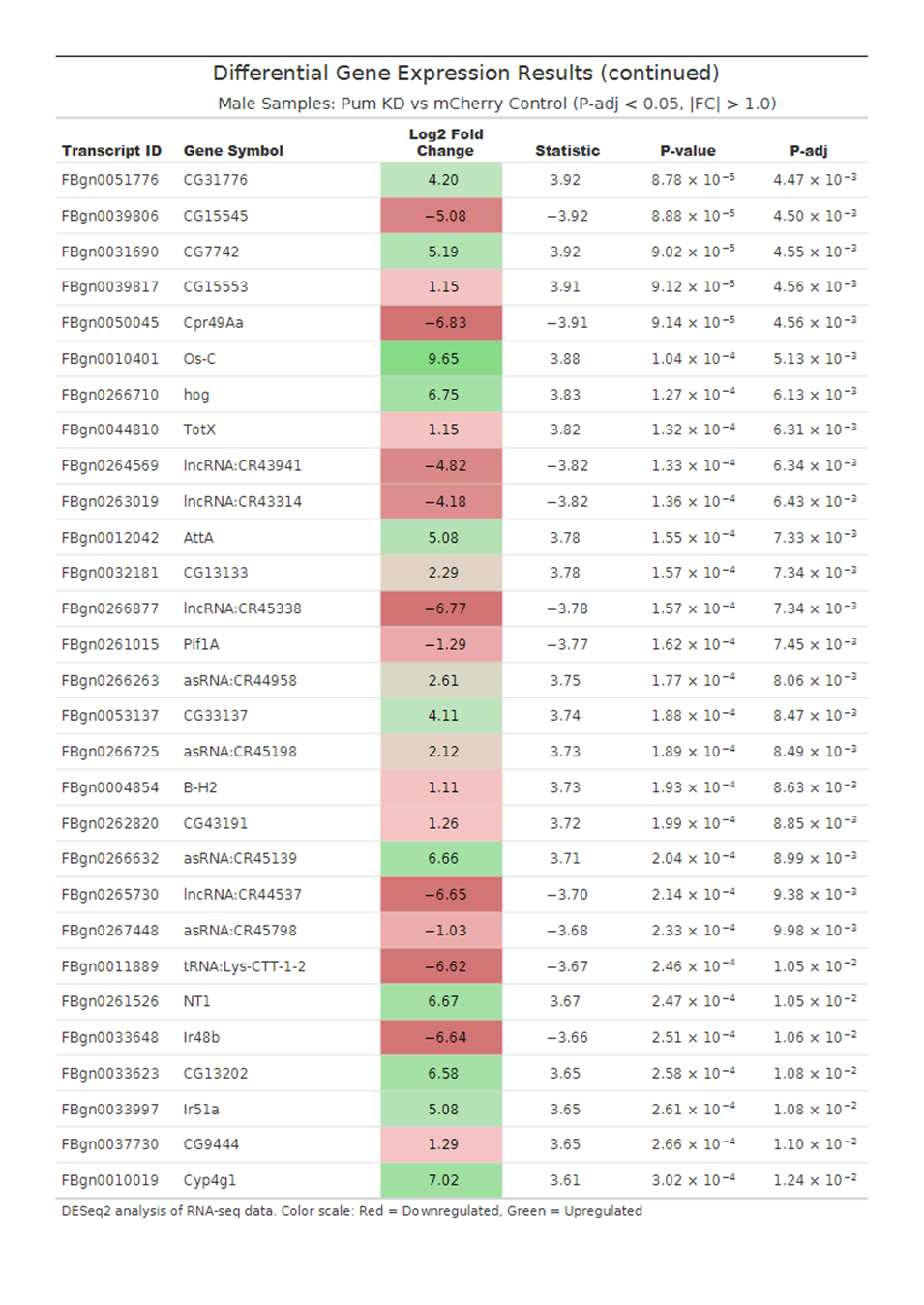

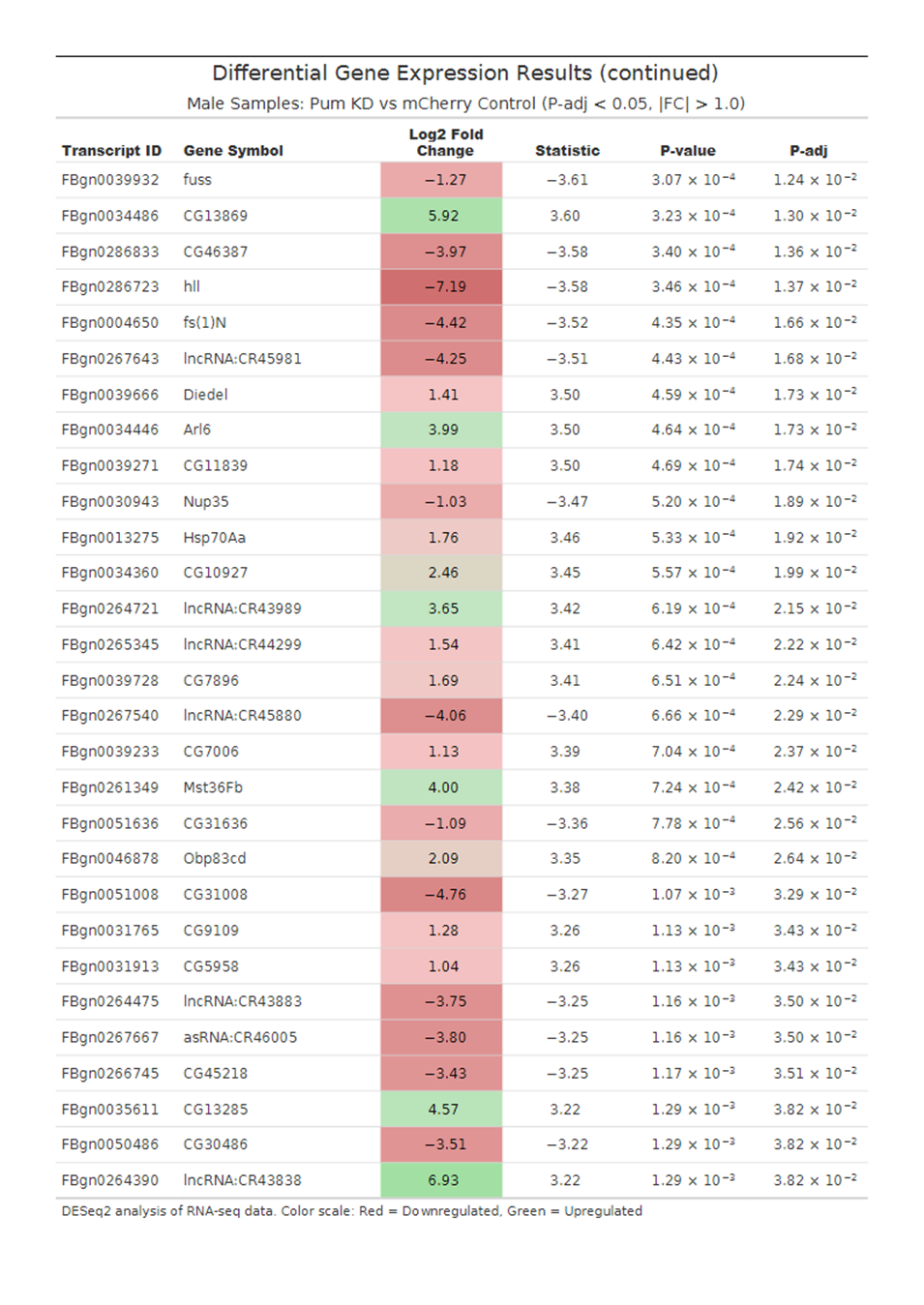

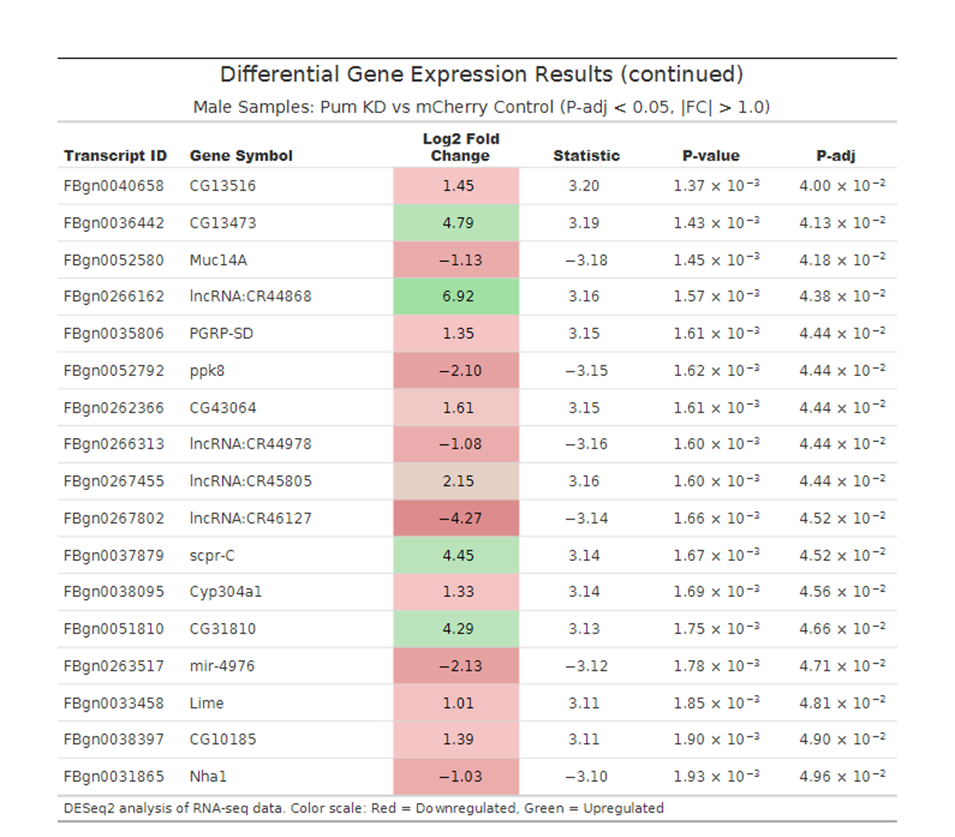


**Table S4. Differentially expressed genes (DEGs) in heads of male flies following peripheral *pumilio* knockdown.** List of the 220 genes found to be significantly differentially expressed (*P*-adj < 0.05, |log_2_FoldChange| ≥ 1.0) in the RNAseq analysis of heads from *esgGal4^ts^, Su(H)Gal80 > UAS-pum^RNAi^* flies compared to *esgGal4^ts^, Su(H)Gal80 > UAS-mCherry^RNAi^* controls. The background color of the log_2_ fold change value indicates upregulation (green) or downregulation (red).
